# Dentistry in Austria

**Published:** 1884-11

**Authors:** Alma Füellgraff


### THE DENTAL REGISTER.

VOL. XXXVIII.] NOVEMBER, 1884. [No. 11.

### COMMUNICATIONS.

### Dentistry in Austria.

BY ALMA FÜELLGRAFF, D.D.S.

It is a critical thing for me to write about dentistry in Austria, as I have to throw a light upon matters that are not of a very agreeable nature. However, my much esteemed friend, the Editor of the REGISTER, suggested that I should write on this subject, and I shall endeavor to acquit myself of this task in the most impartial way possible. My professional brothers in America are well acquainted with the excellency of the German and Austrian Universities, and are also aware that every young man (ladies are excluded) who applies for admittance, has to stand a rigorous examination at the high school, which he is required to attend for eight years, before he is entitled to enter the university. The law requires five years study at the university, the course, however, is seldom completed in less than six years. They know also that no one can practice medicine without having passed several examinations at the university, which are divided into practical and theoretical; and the last, the so-called State examination, is by no means easy. By such proceedings, the public is pretty well preserved from quacks, and can have, and usually has the utmost confidence in their physicians. For dentistry, this step-child of medicine, there is no chair, at the university. Young men who wish to become dentists must work for some years in a dental office, and learn the practical part of their profession, either from some one who has been formerly a barber and bone setter, and whose chief ability consists in extracting teeth with a key, as well as with the forceps, occasionally breaking the wrong one, or a tooth that would have been of service to the patient for a lifetime, had it been properly treated; or they must learn from a physician who may be well posted in medicine, but as to his knowledge in dentistry, I may use the proverb, "hears the bell ring, but does not know where it is hanging."

In fact, as excellent as the German and Austrian universities are for the study of medicine, as poorly are they provided for the study of dentistry. At almost every university there are some so-called lecturers who give voluntary lectures, for which they receive no compensation, and which the students may attend or not, as they choose. Medical doctors are alone entitled to practice dentistry in Austria, and strange to say, these dentists do not explain to their patients or professional brothers of what immense value to the entire human system it is to save the teeth. Free trade has somewhat improved matters in Germany, and has given the Americans a chance to practice. They bring new views, new ideas, and excellent work to a nation, who bye and bye becomes acquainted with the value of it. Their German professional brothers, however, do their best to make life a burden to their American colleagues, out of professional envy. No German, or in fact any foreigner, is allowed to put "dentist" on his sign, unless he has graduated in dentistry in Germany, nor can he have his name as such in the directory, or advertise in the papers, unless he is fond enough of the Commonwealth to be anxious to pay an extra contribution in order to help arranging the national funds. In Austria there is no free trade, consequently no chance for an American to practice in this beautiful country. Dentistry is yet a child in Europe, a weak child; in Austria, it is still in its swaddling clothes; a next natural consequence of the law which nevertheless was not able to protect a score of so-called "technikers," who practice dentistry "illegally," who generally have no scientific education in dentistry or in any other branch. In Vienna there are 280 dentists, the "technikers" included, there are also a few dentists who have studied for a time in Philadelphia, or in some other place in America, and have associated themselves with some one (a physician, probably, who has failed to make his way), whose sign they have put at their doors, and under whose wings the dentist may take shelter, in case any discontented or ill-disposed patient makes a fuss. For woe to the dentist of this class who makes a mistake, for instance putting on a pivot tooth, and the patient has a swollen face afterward, he or she may run to a legal dentist, and a score of these will raise their virtuous voices and implore the liberal laws to see to the rights of the ill-treated patients. It is clearly seen that the existence of these dentists is spent in fear and trembling, and it is also evident that dentistry is by no means in the proper hands. In fact killing the nerve is the great war cry of the dentists of this country, and the public not being enlightened, are contented with this mode of treatment, the more so, as it did not hurt when doctor so and so filled the tooth. In fact the public are cowards when anything is to be done to their teeth, not excepting the bravest soldiers that stood like a wall in the most fierce combat. With a few exceptions the dentists of this country have no idea of treating the pulp, and most of them have not a notion of gold fillings. The patients generally avoid going to a dentist until the tooth is already involved in disease, and the pain has become unbearable. The first thing the dentist does is of course to kill the nerve, and sometimes the poor nerve protests against being killed, and the patient has to call for days and days (heaven only knows how the dentist manages) until the obstinate nerve gets the worst of it. Then he cleans the cavity by a few scrapes of the excavator, and fills it with amalgam or some kind of cement, and the thing is done. Is such plastering work worthy the name of dentistry? I repeat, there are exceptions, but this is the rule. Some dentists make pretty good amalgam fillings, but very few are able to make a gold one, as the Americans do, and they certainly do not know how to make a difficult gold filling. They use gold only where it is easy to manage. As to plate work, which is a great deal better understood, some make even very handsome work, but on the average, it cannot be compared to the American. Altogether it is a sad state of affairs, and so long as the Austrian laws exclude foreigners, and particularly Americans, from the practice of dentistry, it will become little or nothing better. I hope, and of course all dentists who love dentistry as a science will hope, that the day when Austria will be enlightened in this matter, may not be far away, and that it will alter its laws for the welfare of its people.

